# Functional Partitioning of Yeast Co-Expression Networks after Genome Duplication

**DOI:** 10.1371/journal.pbio.0040109

**Published:** 2006-04-04

**Authors:** Gavin C Conant, Kenneth H Wolfe

**Affiliations:** **1**Department of Genetics, Trinity College, University of Dublin, Dublin, Ireland; University of BathUnited Kingdom

## Abstract

Several species of yeast, including the baker's yeast *Saccharomyces cerevisiae,* underwent a genome duplication roughly 100 million years ago. We analyze genetic networks whose members were involved in this duplication. Many networks show detectable redundancy and strong asymmetry in their interactions. For networks of co-expressed genes, we find evidence for network partitioning whereby the paralogs appear to have formed two relatively independent subnetworks from the ancestral network. We simulate the degeneration of networks after duplication and find that a model wherein the rate of interaction loss depends on the “neighborliness” of the interacting genes produces networks with parameters similar to those seen in the real partitioned networks. We propose that the rationalization of network structure through the loss of pair-wise gene interactions after genome duplication provides a mechanism for the creation of semi-independent daughter networks through the division of ancestral functions between these daughter networks.

## Abstract 2

An analysis of how duplicated networks of genes (as a result of whole genome duplication in yeast) evolved shows that network partitioning occurred through loss of interactions, resulting in independent subnetworks.

## Introduction

Beyond its obvious potential for creating new gene products [ 1], gene duplication also affects the structure of genetic networks [ 2– 4]. Duplication initially increases the number of network interactions, but the subsequent loss of interactions can give rise to networks with novel architectures. The particular changes will depend on the type of duplication: i.e., single gene duplication versus segmental or whole genome duplication. Here we study network evolution after a whole genome duplication in the yeast Saccharomyces cerevisiae [ 5, 6].

**Figure 1:**
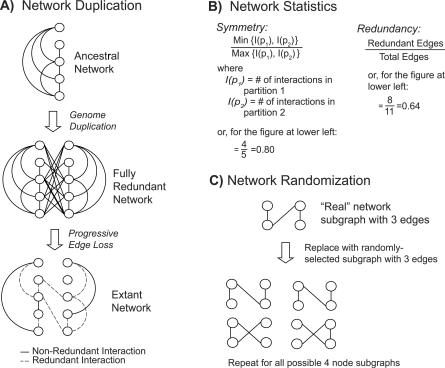
Network Duplication (A) A view of network duplication illustrating our representation of these networks. Nodes (genes) directly opposite each other are paralogs resulting from WGD. Given genes *n _1_* and *n _2_* and their respective paralogs *p _1_* and *p _2_,* redundant interactions (dashed lines) are those that occur more than once in the set *n _1_*: *n _2_, n _1_*: *p _2_, p _1_*: *n _2_*, and *p _1_*: *p _2_*. (B) Two statistics used to quantify these networks. Symmetry measures the degree to which one network partition has more interactions than the other. Redundancy measures the proportion of edges which have survived in more than one copy since duplication (see main text). Example values for the extant network in (A) are also given. (C) Network randomization via subgraph replacement. Each quartet of nodes is randomly replaced by one of the possible subgraphs with the same number of edges. Subgraph frequencies are dependent on the overall edge frequencies of the nodes.

Previous studies of network evolution have not needed to differentiate between single-gene and whole genome duplication [ 2– 4, 7]. However, genome duplications are interesting because they provide networks with many simultaneously duplicated nodes. After such an event, the number of genes (nodes) in the network has doubled, while the number of interactions has quadrupled ( Figure 1A) [ 8, 9]. Subsequent interaction gain or loss reduces redundancy [ 8], generally rapidly [ 10– 12].

We searched for patterns in how surviving interactions are partitioned among the duplicate genes. In particular, it is possible that specialization among the duplicates would yield a network divided into two parts, each having one copy of each pair of paralogs [ 13– 15]. An interesting example of this possibility involving the apparent duplication of the glucosinolate synthesis pathway in *Arabidopsis* has been identified by Gachon et al. [ 16]. After such specialization, we would expect that interactions between genes would be mostly confined within the two new subnetworks with few interactions crossing between them. Another example of this process, discussed below, concerns the glucose metabolism pathway in yeast. This pathway contains several duplicate gene pairs from whole-genome duplication (WGD) that are active under differing cellular conditions. These include genes for glucose sensing *(SNF3* and *RGT2),* glucose transport *(HXT6/HXT1)* and the enzymes that catalyze the initial reaction of glycolysis (hexokinases *HXK1* and *HXK2*). In all three cases, the first member of the pair is involved in the metabolism of glucose at lower concentrations than is the second member [ 17– 19]. The idea that gene paralogs formed at WGD can associate into semi-autonomous subnetworks can be thought of as the “division of labor” over evolutionary time, with duplicate pairs specializing in a particular part of the ancestral network. It is also closely related to models of duplicate gene divergence through subfunctionalization [ 20, 21].

We have studied the evolution of a subset of the yeast genetic network containing the 551 gene duplicate pairs preserved since the whole genome duplication [ 5, 22]. First, we show that such networks tend to display asymmetry and redundancy in their interaction distributions. We next present evidence that some of these networks have significant functional partitioning, with concomitant effects on the patterns of protein localization and gene expression. Examples of the genes found in such networks are discussed. Finally, we present models of network evolution that mimic several properties of the real networks and hence provide insight into the forces that may have driven that evolution.

### Central Idea and Algorithm

We represent the paralogs from WGD as a graph divided into two columns with paralogs opposite each other. The order of paralogous pairs in the columns is arbitrary ( Figure 1A). Gene interaction data is overlaid as graph edges and can include protein-protein interactions, shared expression patterns, or interactions between transcription factors and their targets.

We define two types of edge: “internal” edges connecting nodes in the same column (arcs or vertical lines in Figure 1A) and “crossing” edges joining nodes in different columns (diagonal lines in Figure 1A). Although we can speak conceptually of biologically interpretable subnetworks (such as a metabolic pathway), in practice our data will generally not give such clear-cut patterns. Our approach is conceptually similar to a pathway alignment algorithm developed by Kelley et al. [ 23] but focuses on a different optimality criterion and is applicable only to the particular case of WGD, both of which allow us to make stronger assumptions regarding the evolution of interactions.

We thus require a measure on these data that allows patterns of network evolution to be studied. The definition we have used is that of a network partition. Given a set of *n* duplicate gene pairs (2 *n* genes), a partition of *n* genes is created by selecting one member from each duplicate pair. This procedure defines the left-hand column in Figure 1A and implicitly defines the complementary right-hand column. There are 2 *^n^* ^−1^ possible unique partitionings of the duplicates. The above suggests an optimality criterion: define the best partitioning of paralogs as the one that minimizes the number of crossing edges. Our heuristic partitioning algorithm is able to optimally partition networks up to a size of 2 *n* = 402 (see Materials and Methods).

## Results

### Network Redundancy

**Figure 2:**
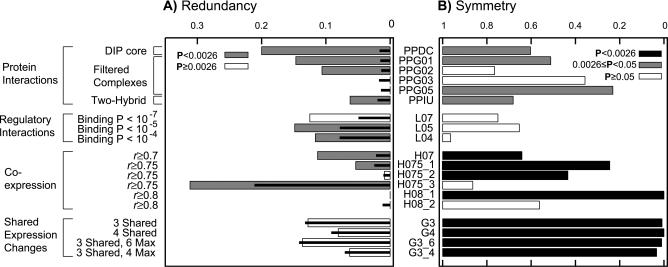
Redundancy and Asymmetry (A) Proportion of total interactions in our networks that are redundant. Black central bars give the mean proportion of redundancy in randomized networks: grey outer bars indicate significant deviation from this expectation ( *p* < 0.002) while white outer bars indicate no significant deviation (Datasets PPG03, PPG05, H08_1, and H08_2 do not show significant differences in redundancy to the randomized networks). (B) Symmetry of inferred partitions for the same 19 datasets. Symmetry is reported as the ratio of the number edges in the edge-poor partition to the number in the edge-rich partition. Cases where the differences from the expectation of *r* = 1.0 were of marginal significance are shown in grey for reference.

Because interactions between genes can be defined at varying stringencies and because not all paralogous pairs interact with other genes, our data naturally presents itself as 19 graph components drawn from six large-scale datasets (see Materials and Methods). These components (containing subsets of the 551 duplicate pairs) will be the “networks” we refer to in our analysis below. Because these networks owe their origins to genome duplication, we searched for redundant interactions (cases where more than one interaction exists between two pairs of duplicates; see Figure 1A and 1B) in the networks. Teichmann and Babu have previously shown in transcriptional regulatory networks that redundant interactions survive even for comparatively ancient duplicates [ 7], so it is reasonable to expect survival of some such interactions since WGD. We compared the proportion of the total network edges that were redundant to the degree of redundancy seen in random networks with the same node degree distribution. Many of the real networks have significantly more redundancy than the random ones, presumably due to genome duplication ( Figure 2A: we apply a Bonferroni correction for 19 hypothesis tests, thus *p* ≤ 0.002, with α < 0.0026 to reject the null hypothesis of no redundancy).

### Network Asymmetry

For each dataset in Table 1 we searched for the optimal partitioning as described above. We then calculated the symmetry between the numbers of interactions in the resulting two partitions. Symmetry *(r)* was defined as the ratio of the number of edges in the edge-poor partition to the number of edges in the edge-rich partition ( Figure 1B). Were edges distributed at random with respect to the partitions, we would expect approximate symmetry ( *r* ≍ 1) between a partition and its complement. Instead, we find for many networks that one partition had significantly more interactions than the other ( *r* < 1.0, two–sided Binomial test with Bonferroni correction, *p* ≤ 2 × 10 ^−5^; Figure 2B). Note that, because we have often used different thresholds to define networks, several of the networks in Figure 2 are subnetworks of other networks (thus networks H075_1, H075_2, H075_3, H08_1, and H08_2 are subnetworks of H07 with higher interaction threshold values of 0.75 or 0.8). This selection procedure will tend to result in networks with some variation in the level of asymmetry (unpublished data).

**Table 1:**
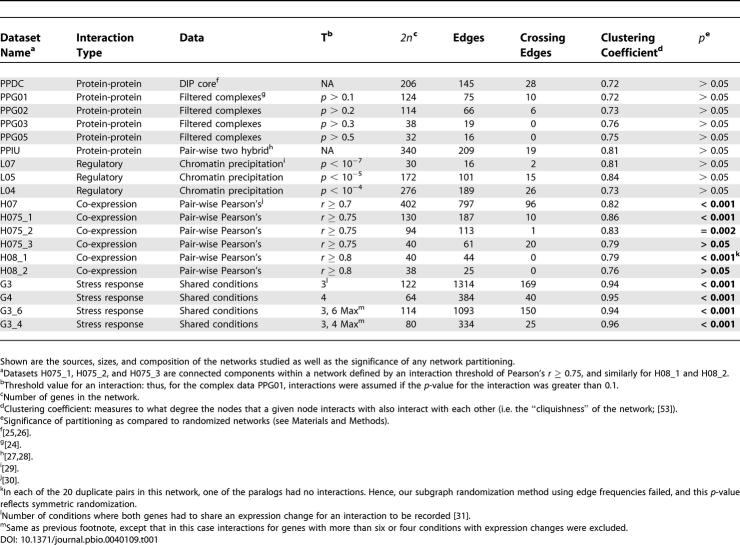
Network Statistics for 19 Analyzed Datasets

### Network Partitioning

As discussed, we searched for evidence for significant interaction partitioning using our algorithm. To determine if the networks showed more partitioning (fewer crossing edges) than we would expect by chance, we randomized the networks and recalculated the optimal partitioning. Randomization was carried out by selecting every possible pair of pair of paralogs (four genes). These four-node subgraphs were replaced at random by another four-node subgraph with the same number of edges (see Figure 1C). The probability of subgraph replacement was made to depend on the inherent asymmetry in interaction degree between paralogs. Thus, we calculated the average fraction *p* of the total number of interactions for a paralog pair that belonged to the interaction-rich paralog. The probability of an interaction joining two interaction-rich genes in a subgraph is then *p ^2^*, while the probability of an interaction joining an interaction-rich gene to an interaction-poor one is 2 *p(1−p)* (because there are two possible interactions of this type). The subgraph replacement probabilities are calculated accordingly.

Neither the protein-protein interaction data [ 24– 28] nor the transcriptional regulatory data [ 29] showed significant network partitioning ( Table 1), whereas there was evidence for significant partitioning in the co-expression data of Hughes et al. [ 30] and the shared expression change data of Gasch et al. [ 31]. For the co-expression data [ 30] we defined interacting genes as those sharing expression similarity (Pearson's *r*) of 0.7 or greater. As Table 1 shows, these data showed significant partitioning of edges when compared to randomized networks ( *p* ≤ 0.002). Of course, the issue of the multiple tests inherent in our approach should be considered. A standard Bonferroni correction is suboptimal for two reasons: first because many of our *p*-values are upper bounds, and second because several of the networks are subnetworks of other networks. We can avoid the second issue by considering only the largest network in each case: we then have six comparisons, with the two gene expression networks still showing significant partitioning after this correction ( *p* < 0.001 for each, with α = 0.008 for the Bonferroni correction). As a further test, we considered the overall distribution of co-expression correlations for the genes in the network H07. As shown in Figure S1, there is a significant difference in the distribution of correlation values between the two partitions and a significantly lower mean correlation for comparing genes between partitions (likelihood ratio test [LRT], unpublished data). Moreover, the 201 duplicate pairs show an average correlation that is significantly higher than the average in either of the partitions, consistent with their recent duplication (LRT, unpublished data). The average correlation between duplicates is nonetheless much lower than our cutoff for an interaction ( *r* = 0.27 as opposed to *r* = 0.7).

**Figure 3:**
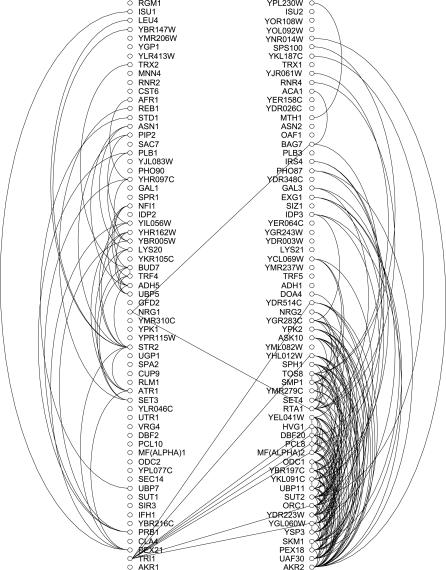
Example Network An example subnetwork (H075_1 in Table 1) with significant partitioning ( *p* < 0.001). Edges join genes with co-expression correlation (Pearson's *r*) ≥ 0.75. There are 65 genes pairs in this network ( *2n* = 130).

An example network (H075_1) is shown in Figure 3. Note the large number of internal edges and the few crossing edges and recall again that WGD paralogs are arranged opposite each other. Hence those genes without interactions are nonetheless of importance: the fact that their paralogs possess interactions indicates divergence between the two genes since WGD. Our results were robust to the exclusion of 55 pairs of ribosomal proteins from the largest subnetwork (H07) and to the random removal of 5% of interactions from this same network (unpublished data).

For the data from Gasch et al. [ 31], who studied the transcriptional response of yeast to 11 stress conditions, we found that because most genes did not show expression changes in most experiments, a correlation analysis similar to that above was inappropriate (many genes were highly correlated with each other because they showed no expression changes). Instead, we considered only genes with a change of expression of at least 3-fold in a stress condition relative to a control condition. Our approach is similar to that used by Wagner [ 32]. We then connected genes that both showed expression changes in the same three (or four) conditions (datasets G3 and G4 in Table 1). A few genes in these experiments showed changes in expression over many of the 11 experimental conditions, and these generalized stress response genes were thus connected to many other genes. To test for any resulting bias, we removed all genes with greater than six (G3_6 dataset) or greater than four (G3_4 dataset) responses, but in both cases the partitioning remained significant ( Table 1). The interaction definition used here can connect gene pairs where one gene is induced and one repressed in a given condition. We feel that such connections are valid because the genes in question show evidence of important associations. However, significant partitioning remains even when only genes whose direction of changes are the same are connected ( *p* < 0.001).

We note that, when the two gene expression analyses (datasets G3 and H07) are combined, roughly 40% of the preserved duplicate gene pairs (230/551) appear in a network showing significant partitioning of interactions.

One would expect network partitioning to leave its mark on other aspects of yeast's cellular organization. We thus examined the distribution of shared regulatory motifs and of protein localization in networks with significant partitioning. We also discuss the concordance of our inferred partitions with a well-understood part of the yeast metabolic network and their association with knockout phenotypes.

### Partitioning, Protein Localization, and Sequence Motifs

An obvious question is whether the pairs of inferred partitions are distinct from one another in where their constituent proteins are located in the cell or in how often different regulatory motifs are found upstream of the genes in question. To test for such differences, we counted the number of proteins located in each of seven subcellular compartments [ 33]. We then asked, using a permutation test, whether the number of genes in each compartment differs between the two partitions inferred for our largest dataset with significant partitioning (H07). An identical analysis was done with a total of 65 sequence motifs [ 34]; see below for details on motifs used. Surprisingly we observe no differences in these two distributions between the partitions (unpublished data). Given our simulation results below, we suggest that, because partitioning appears to be a function of local network structure, the partitions, which consist of many duplicate genes (201), may still be grossly similar at this more global level.

Given than our partitions did not appear to differ in global motif usage or in overall localization distribution, we next considered whether the partitions considered individually showed an excess of pairs of genes located in the same cellular compartment or with the same upstream motif compared to the set of 1,102 genes with WGD paralogs taken as a whole. This test will indicate if the partitions are enriched in functionally related genes. For the two largest networks with significant partitioning (H07 and G3), we tested whether the partitions had more co-localized proteins (i.e. proteins found in the same cellular compartment; [ 33]) than we would expect given the overall frequency of co-localization among these 1,102 genes. In both cases the partitions showed a significant increase in co-localization ( *p* < 10 ^−5^). We further considered whether the identified network partitions were associated with DNA-level sequence motifs by studying the frequency of 71 conserved sequence motifs identified by Kellis et al. [ 34] in the 1,500 basepairs upstream regions of duplicate gene pairs. For these same two networks (H07 and G3), we first compared the average number of shared motifs for pairs of genes within each partition (λ *_p_*, fit to a Poisson distribution using maximum likelihood; see Figure S2) to the average number of shared motifs for a sample of 20,000 random pairs of the 1,102 genes (λ *_r_*). We also compared the values of λ *_p_* between each pair of partitions. In these networks both partitions had λ *_p_* > λ *_r_* ( *p* ≤ 0.0003; LRT). We also saw that one partition always had significantly more shared motifs than other partition ( *p* ≤ 0.002; LRT). Our results indicate that the differences in gene expression patterns seen between partitions are mirrored by differences in sequence-level motifs. This result is perhaps not surprising as these motifs likely play a role in regulating expression.

### Partitioning and Cellular Metabolism

We have already discussed the idea of functional partitioning of gene pairs after duplication. With apologies to Adam Smith, we refer to this possibility as the division of labor, with the implication that it allows the functional specialization of duplicate genes. A known example of this concerns a WGD duplicate pair involved in glycolysis but not found in networks H07 or G3. The proteins encoded by the genes *CDC19* and *PYK2* both catalyze the last reaction in glycolysis, the conversion of phosophoenol-pyruvate to pyruvate. However, *CDC19* is induced by the upstream metabolic intermediate fructose-1–6-biphosphate, while *PYK2* is not [ 35]. This difference can be understood if it is noted that *PYK2* is active at lower glucose concentrations than is *CDC19* [ 35], conditions where the concentration of upstream metabolites may be insufficient to induce the required activity. Thus, this duplication frees the cell from having to make a trade-off between efficiency at high and at low glucose levels. Similar divisions are apparent in our networks: two high-affinity hexose transporters, *HXT4* and *HXT6,* are both placed in the same partition as the hexokinase gene *HXK1* in networks H07 and H075_2, in agreement with the known roles of these genes in low-concentration glucose metabolism [ 17– 19]. Similarly *HXK1* has many interactions in stress response network G3, while its WGD paralog *HXK2,* active during “standard” conditions of glucose fermentation, does not. Functional differences also exist between *HXK1* and *HXK2*. In particular, *HXK2* has regulatory functions including the regulation of its paralog *HXK1* [ 19, 36, 37]. Considered together, we believe the above facts constitute evidence for division of labor, with one group of glycolytic gene duplicates functioning at low glucose levels and the other at higher levels.

In addition, the co-expression networks (H07 and H075_2) also contain some smaller gene “circuits” which may be associated with responses to growth in the absence of glucose. The duplicate gene *SIP3* (WGD paralog: YHR155W) is part of the SNF1-complex responsible for inducing glucose-repressed genes in the absence of glucose [ 38, 39]. Several of the genes *SIP3* interacts with fit plausibly into such a regulation pattern. These include the glycerol transporter *GUP1* [ 40], the glyoxalase *GLO2* [ 41], and the gene *DCI1* involved in fatty acid oxidation [ 42], all genes whose metabolic activity would need to change depending on glucose levels. The paralogs of these three genes either show different phenotypes *(GUP2, ECI1)* or are only present in the mitochondria *(GLO4),* suggesting functional divergence.

It is important to bear in mind that despite the attractiveness of the hypothesis of complete network duplication followed by specialization, the high levels of gene loss observed in yeast after WGD [ 43] will hamper our ability to perceive biologically relevant patterns in these data (because the resulting single copy genes that form part of any complete biologically relevant network are not present in our analysis).

### Stress Response and Knockout Phenotypes

Given the high asymmetry in the stress response datasets, it is natural to ask what the role of the interaction-poor paralogs is. To do so, we examined gene knockout data from Giaever et al. and Steinmetz et al. [ 44, 45] (curated by SGD; [ 46]). For the largest stress response network (G3) there is no significant difference between the number of interaction-rich paralogs which have detectable knockout phenotypes when their paralogs do not and the number of interaction-poor paralogs who have such phenotypes (nine versus eight). This result argues against the interaction-rich paralogs being generally more important. Coupled with the above results, there are thus strong indicators of differences in gene function between these duplicate genes.

### Simulated Network Evolution

**Figure 4:**
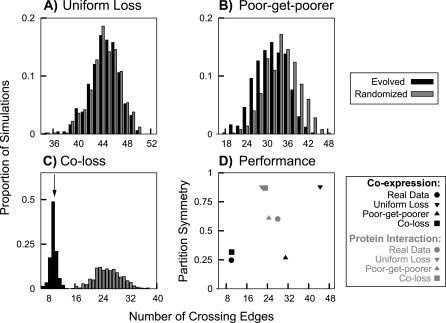
Modeling Interaction Loss (A–C) Three models of network interaction. Black bars are the number of crossing edges in the simulated networks. Grey bars are that number after randomization. (D) compares the symmetry and number of crossing edges of the real data to the averages from the various models. For (D), results of simulations carried out on the DIP core set of protein interactions (dataset PPDC in Table 1) are shown in grey for reference (see main text).

Given the existence of networks with significant partitioning, we used simulations of network evolution to help us understand the forces at work. Starting from a fully-redundant network derived from dataset H075_1 ( Figure 3), we simulated network evolution under three models of interaction loss (see Materials and Methods) and examined the distributions of crossing edges seen among 500 simulation replicates (black bars in Figure 4). In the simplest model (“uniform loss;” Figure 4A), interactions are lost at random until redundancy reaches the level seen in the real network. The second model (“poor-get-poorer;” Figure 4B) makes the probability of interaction loss inversely proportional to the number of ancestral interactions retained by the two nodes involved. The third model: “co-loss” ( Figure 4C), makes the probability of edge loss dependent on the number of shared neighboring nodes *S* of the two nodes *n _1_* and *n _2_* (i.e. the number of nodes with which both *n _1_* and *n _2_* have an interaction). We compare this to *S _max_*, the maximum number of shared neighbors across the four combinations of these two genes and their respective paralogs *p _1_* and *p _2_* (i.e., *n _1_:n _2_, n _1_:p _2_, p _1_:n _2_,* and *p _1_:p _2_*). Edge loss probability decreases with increasing *S/S _max_*.

The performance of the three models was assessed by comparing the degree of partitioning seen in the 500 simulated networks (black bars, Figure 4A–C) to the partitioning seen after randomizing each of these simulated networks using the above subgraph replacement approach (grey bars). If these two distributions are similar that suggests that that model does not produce partitioned networks. The second two models ( Figure 4B and C) have a variable parameter allowing us to tune the simulations to be similar to the real networks. For Figure 4B, separation between the simulated networks and their randomized counterparts is low, but asymmetry approaches values seen in the real data ( Figure 4D). In Figure 4C, tuning the model gave an average proportion of crossing edges nearly identical to the real data (10.11 versus 10; arrow in Figure 4C). Also, although mean symmetry in these simulations ( Figure 4D) was higher than in the real co-expression data, median symmetry was lower. Thus, the co-loss model ( Figure 4C) provides a close approximation to real data for two parameters and gives clear separation between the simulated networks before and after randomization.

For comparative purposes, we performed similar simulations on a network without significant partitioning, the protein-protein interactions from the database of interacting proteins (DIP core, dataset PPDC in Table 1). In this case the co-loss model did not generate significant partitioning or asymmetry ( Figure 4D), likely because the input network lacked the clustering needed for such patterns to emerge (note the lower clustering coefficients for this network as compared to H075_1 in Table 1). The poor-get-poorer model instead provided the best fit to this network. Asymmetry was very similar to the real data, and the number of crossing edges in the simulations was not significantly different from the actual network (unpublished data). We discuss implications of these simulation results and of the division of labor among duplicates more generally below.

## Discussion

Our analysis of the pattern of gene interactions seen among duplicate genes in S. cerevisiae reveals interesting high-level features of network duplication. There has been considerable, though incomplete, loss of redundant interactions since WGD, as well as development of significant asymmetry in interactions in several cases ( Figure 2).

We also found evidence for the partitioning of network interactions between duplicate genes in gene expression networks. Because partitions are inferred algorithmically, it is important to be certain they have biological significance. We point to three distinct lines of evidence that this is the case. First, real networks possess more partitioning than do randomized ones. Second, partitions show a non-random distribution of shared regulatory motifs. We would not expect this were the partitions biologically irrelevant. Finally, at least some partitions show differences in the frequency of protein co-localization.

Van Noort, Snel, and Huynen have previously observed that gene co-expression networks can evolve in a modular fashion, whereby, after the shared duplication of an ancestral pair of co-expressed genes, the two pairs of paralogs diverge in expression to form two new genetic “circuits.” Each circuit of two co-expressed genes contains one member of each duplicate pair but the circuits are not themselves co-expressed with each other [ 15]. Our analysis allows us to study such patterns at a global scale, rather than focusing on pair-wise comparisons. Moreover, because our set of duplicate genes is known to have originated with a single event, we can make stronger conclusions regarding the evolution of the network. We thus find larger scale examples of this phenomenon, with several pairs of duplicates diverging into parallel functional groups (such as members of the glycolytic pathway). However, as Figure 3 indicates, patterns of network evolution are more complex that simple pair-wise pathway divergence.

The obvious question raised by our analysis is whether the partitioned networks we observe formed through the degenerative partial loss of ancestral functions (subfunctionalization; [ 20, 47, 48]) or the appearance of new functions (neofunctionalization). For the glycolytic genes, it seems plausible to argue for the former, as the ancestral yeast certainly expressed the glycolytic pathway genes at both high and low glucose levels. However, because we lack detailed knowledge of the regulation involved, we cannot make this claim absolutely. This difficulty is a general one, and, if we have a slight preference for the subfunctionalization hypothesis, it is simply because it is the more parsimonious one.

Our simulations of network evolution also provide some insight into these questions. We were able to generate networks with partitioning similar to real data, based on a set of rules that make interaction loss more or less favorable depending on the local interaction environment of a duplicate ( Figure 4C).

Our three models allow interactions to be lost through genetic drift or directional selection and to be maintained by purifying selection. The models differ in how “knowledge” of the total network is allowed to influence selection. Under the uniform loss model, nodes have no knowledge of the wider network, implying that a gene's function is independent of other genes. Given these features, it is unsurprising that this model produces symmetric, non-partitioned networks ( Figure 4A) which are most similar to the regulatory networks studied (which also show neither partitioning nor asymmetry). One can argue that the loss of a single binding site for a transcription factor may have a relatively limited impact on whether that factor will lose other interactions, which may be the reason for the similarity between these simulated networks and the regulatory data. The poor-get-poorer model allows local knowledge to affect interaction retention. The result is asymmetric but non-partitioned networks similar to real protein-interaction networks (which exhibit weak asymmetry in their interactions, see Figures 2 and 4D). This similarity implies that loss of a direct protein interaction would be disadvantageous but that the loss of a distant interaction in a partner protein would have a much weaker effect. This conclusion is supported by the fact that the poor-get-poorer model created simulated networks with interaction patterns very similar to the protein-protein interaction network used to seed the simulations ( Figure 4D). The co-loss model incorporates regional knowledge by considering how many shared neighbors two interacting genes have. Here, a gene's effectiveness depends both on direct partners and on more distant connections. Note that the nature of co-expression network evolution is inherently regional, because changes in one gene's expression pattern could simultaneously disrupt its expression correlation (and hence “interaction”) with several other genes. Thus, it is not unexpected that the co-loss model gives rise to networks similar to real co-expression networks. Partitioning arises under this model because we require all ancestral edges to be preserved in at least one copy. Thus, interaction loss in one paralog will naturally give rise to a subnetwork containing that gene's paralog which preserves the relevant interactions. Finally, it is interesting to note that the co-loss model can create partitioning by a process that is strictly degenerative and hence similar to the subfunctionalization model of Force et al. [ 20]. Such a possibility belies the idea that all complexity in living systems must evolve through directional selection,

The ramifications of how genome duplication may lead to the division of labor among duplicates needs further exploration. Although it increases the complexity of a system without any necessary improvement in function, the new network layout may have other desirable features such as robustness [ 49] or evolvability [ 50]. Moreover, the presence or absence of partitioning in the network may be indicative of the internal dependencies of the nodes upon each other. All of these ideas will be interesting possibilities to test with future functional genomic data.

## Materials and Methods

### Partitioning algorithm

We first use a greedy search (sequential addition of paralogs minimizing the number of added crossing edges at each step) followed by local pair-wise exchanges to identify a candidate solution with few crossing edges. Using this candidate solution, we recursively search a binary tree of all possible permutations. We apply a branch and bound approach, such that at any internal node in this tree we have added *i* paralog pairs to a “family” of potential permutations (thus if *n* = 4, one internal node of this tree at depth *i* = 3 would have the form 010×, where × can take on values of either 1 or 0). If the number of edges crossing in the permutation family is as large as the number seen in the best permutation so far, evaluation of that permutation family can be abandoned, as the score of any permutation in that family can be no better than the best score so far. Every such permutation family abandoned saves a total of 2 *^n−i^* ^−1^ permutations which need not be considered. To make this *i* as small as possible, our algorithm adds node pairs with high degree first, which causes the score to climb quickly in the initial branchings.

We save further time by noting that genes with interactions that connect to both of a pair of paralogs must add one crossing edge to the score for any permutation. We maintain a lookup table of such instances, allowing us to determine if the final score of a partition family will exceed the current best score and so to abandon that permutation.

The performance of this algorithm is such that we were able to analyze a network of *n* = 201 (roughly 10 ^60^ permutations) in 73 s on a 3-GHz Pentium 4 Xeon.

### Data sources

A total of 551 gene duplicates previously described as owing their origins to the whole genome duplication in yeast were obtained from the Yeast Gene Order Browser project [ 22]. All gene names used in the text and figures are taken from the *Saccharomyces* Genome Database [ 46]: sequences and systematic names can be obtained from this source. Duplicate identification was made based on shared gene order across several species of yeast, both with and without the genome duplication. A list of these gene pairs is available at http://wolfe.gen.tcd.ie/ygob/doc/Byrne_Supp_Table2.xls.

Protein interaction data were obtained from: 1) a filtered dataset of highly supported interactions from the DIP core [ 25, 26]; 2) an analysis by Gilchrist, Salter, and Wagner [ 24]; and 3) by pooling pair-wise protein interactions from the two-hybrid experiments of Ito et al. [ 27] and Uetz et al. [ 28]. Transcription factor binding data were taken from the results of Lee et al. [ 29] and filtered on their reported *p*-values. Expression data were obtained from the expression compendia of Hughes et al. [ 30] and the stress response microarray experiments of Gasch et al. [ 31]. For the data of Hughes et al. (hereafter “co-expression data”), we calculated, for each pair of genes, the Pearson's correlation coefficient *(r)* between the two genes. Only gene pairs for which both genes shared measured expression levels for at least 200 experiments were considered. The data of Gasch et al. reports the response of yeast cells to a number of stress factors. Following Wagner [ 32], we considered data for 11 different stress conditions: heat and cold shock, oxidative stress, treatment with menadione, diamide, or dithiothrietol (DTT), hyper and hypo-osmotic stress, amino acid and nitrogen starvation, and cells in stationary phase cultures. For each gene and experiment, the absolute value of the maximal expression change (induction or repression) was found. We refer to these data hereafter as “shared expression changes.”

To assess whether cross-reactivity in the above assays was likely to confound our analysis, we examined the synonymous divergence of the 551 genes pairs. For the 19 networks in Table 1, only network H07 had more than ten paralog pairs with a pair-wise K _s_ < 0.2. Subsequent analysis revealed that all but four of the 21 pairs with K _s_ < 0.2 in this network were ribosomal proteins. For this reason we repeated the analysis of network H07 excluding ribosomal proteins.

### Graph components

Because our algorithm assumes that all paralog pairs have at least one connection to another gene in the network, it is properly applied to connected components within a graph. When identifying these components, we required that members of a duplicate pair always be in the same component.

### Network randomization

Network randomization was carried out as described in the main text (also see Figure 1C). A total of 100 initial randomizations were performed. For cases where significant partitioning was identified ( *p* < 0.05), a further 1,000 randomizations were performed ( Table 1).

### Network asymmetry

To detect asymmetry in the partitions ( Figure 1B) we compared the observed symmetry *r* between partitions to the expectation of *r* = 1.0. Our approach might incorrectly infer significant asymmetry if the partitioning algorithm tended to group interaction-rich genes into the same partition. To be sure we were not so mislead, we randomized the networks using subgraph replacement under an assumption of symmetric edge distributions, generating symmetric random networks. We then compared the symmetry values for 100 of these simulations to the values from the real networks. In all cases, the *p*-value from this approach was in close agreement with those in Figure 2B.

### Analysis of largest co-expression network

Randomization of our largest network (H07) resulted in irregular new networks that our algorithm could not optimally partition. Instead we used simulated annealing to find the best partition of the randomized networks in this case (the real network was easily solved by our exact algorithm due to its ordered structure). For each of 1,000 random networks, ten simulated annealing runs were made. In all cases, at least two runs resulted in the same lowest score. The best score found among these 1,000 random graphs (156 crossing edges) is 1.5× larger than the score in the real data (96 crossing edges).

### Co-localization

We computed what proportion *p* of all possible pairs of the 1,102 paralogs were co-localized in our data ( *p* = 0.09). Using a binomial test, we compared this proportion *p* to the proportion of co-localized genes seen in the various partitions.

### Motifs

We examined the density of shared DNA-sequence motifs in our two largest networks showing significant partitioning (H07 and G3: other networks with significant partitioning are subsets of these two). Using 71 motifs identified by Kellis et al. from S. cerevisiae and three closely-related species [ 34], we searched the 1,500 base pairs upstream of the start codon of each gene of interest. One of the motifs identified by Kellis et al. was excluded because of its variable length. We required exact matching across all motifs, a conservative approach that should not bias our analysis of relative motif density between partitions or between partitions and random genes. We also analyzed our matches after excluding the two most common motifs.

### Simulation of network evolution

We proposed simple models of network evolution to compare to the real data. We chose to evolve networks to achieve the same redundancy as an extant network. Thus, after duplication, the network evolves strictly by loss of interactions. Although this is clearly over-simplistic [ 11], we note that previous work has suggested that loss of network interactions after duplication is indeed more common than gain [ 10].

Simulations were initiated with a fully redundant network (i.e. all edges present in four copies). All of our models then iterate over the number of remaining redundant edges until that number is equal to that seen in the input network. At each step, the total probability of edge loss is scaled to 1.0 and a uniform random number on this interval is used to select the edge to be lost. The models differ in the probability of loss assigned to a given redundant edge.

### Uniform loss

This simple null model makes the probability of loss of any redundant edge equal to that of any other redundant edge. We use this model as a basis of comparison to the more complex models below.

### Poor-get-poorer

Several types of biological networks show a power-law scaling of interaction degree (e.g. protein-protein interaction [ 10] and metabolic networks [ 51]). For such networks, the preferential attachment of new interactions to nodes with many existing interactions is critical to yielding this degree distribution [ 52]. By analogy to this “rich-get-richer” phenomena, we propose a “poor-get-poorer” mode of interaction loss after duplication. For two nodes *i* and *j* sharing an edge, the probability of the loss of that edge scales as:



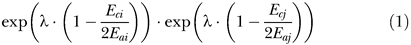



where *E _ax_* is the number of edges seen in the corresponding ancestral node ( *x* = *i,j*) and *E _cx_* is the current number of edges for node *x.* Here, λ is a scalable parameter greater than 0. Thus, when all edges have been retained, we have a minimal value of this expression: exp(λ·0) = 1.0.

### Co-loss

The final model attempts to incorporate some regional properties into the loss of interactions (see above). One way to do this is to make the loss probabilities depend on the neighbors of each node. Thus, we make edge loss scale as:



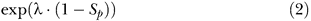



where *S _p_* is given by



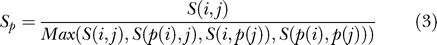



*S* is the number of other nodes *k* which are connected to both *i* and *j,* and *p(x)* gives the paralog of node *x* in our dataset.

## Supporting Information

Figure S1 Distribution of Pair-Wise Expression Correlations for the Genes in the Dataset H07 Plotted are four distributions: those for pairs of genes within each group (“Group 1,” “Group 2”), one for pairs of genes where one member of the pair is in Group 1 and the other in Group 2 (“Cross-group”), and one for the 201 duplicate gene pairs in this dataset (“Dupl. Pairs”). The means for the four distributions are 0.054, 0.015, 0.002, and 0.271, respectively. Note that Group 1 in particular is enriched with high correlation values (the blue “hump” on the right of the distribution), whereas the cross-group distribution has a mean closest to zero, indicating little enrichment of co-expressed genes, but rather merely the random correlation value of 0.0, which would be expected. The duplicate gene pairs from WGD show higher correlations, which is presumably an indication of their recent common ancestry.
(1.2 MB EPS)

Figure S2 Density of Shared Motifs among Two Pairs of Inferred Partitions Values of λ on the *y*-axis are the average number of shared motifs per gene pair estimated by maximum likelihood for the two partitions (λ *_p_* in the text) and for random pairs of genes (λ *_r_* in the text). For each of the datasets ( *x*-axis) two values are given: the density of shared motifs in the motif-poor partition (light bars) and the density of shared motifs in the motif-rich partition (dark bars). The black line indicates the density of shared motifs in pairs of genes drawn at random from our *2n* = 1,102 genes of interest. Vertical black bars connecting to this line indicate a significant difference from the random value.
(A) All motifs used. (B) The two motifs with highest frequency excluded.
(1.2 MB EPS)

## Abbreviations

DIP: database of interacting proteins
LRT: likelihood ratio test
WGD: whole-genome duplication
